# Molecular identification of nontuberculous mycobacteria using the *rpoB*, *argH* and *cya* genes analysis

**DOI:** 10.1186/s13568-022-01463-1

**Published:** 2022-09-19

**Authors:** Azar Dokht Khosravi, Mohammad Hashemzadeh, Parisa Rokhfirooz

**Affiliations:** 1 Infectious and Tropical Diseases Research Center, Health Research Institute, Ahvaz Jundishapur University of Medical Sciences, Ahvaz, Iran; 2 Department of Microbiology, Faculty of Medicine, Ahvaz Jundishapur University of Medical Sciences, Ahvaz, Iran; 3 Iranian Group On Microbial Drug Resistance, Tehran, Iran

**Keywords:** Mycobacteria, Nontuberculous mycobacteria, Molecular identification, *rpoB* sequencing, *argH*, *Cya*

## Abstract

Nontuberculous mycobacterial (NTM) infections are growing worldwide especially in immunocompromised individuals. Since treatment of NTM infections is species-specific, the precise identification of NTM to species level is critical for an optimal treatment. This study was aimed to identify different NTM species by sequencing the *rpoB* gene and evaluating the effectiveness of *argH* and *cya* gene markers. In total 64 clinical isolates suspected to NTM were collected. The identification of the isolates was done by standard conventional methods and PCR-based *rpoB* gene and sequence analysis. PCR sequencing of *argH* and *cya* genes was performed to evaluate the efficacy of these genes in identifying and differentiating different species and subspecies of NTM. Among 64 isolates tested, 51 (79.68%) were detected by conventional tests as NTM. The results of *rpoB* sequence analysis revealed that the 56 clinical isolates were identified in 10 species of NTM and 8 remaining isolates which showed ambiguous results by *rpoB* sequencing, application of *argH* and *cya* sequencing could detect these isolates. Furthermore, by using *cya* gene sequencing, *M. abscessus* subspecies were properly differentiated. Although the *rpoB* sequencing as a standard method, is beneficial for detecting various species of NTM, however, based on our findings, *argH* and *cya* gene markers have a superb ability to discriminate closely related species. Further investigations are required to verify our outcomes.

## Introduction

Nontuberculous mycobacteria (NTM) are a collective name referring to a group of environmental organisms, apart from *Mycobacterium tuberculosis* (MTB) and *Mycobacterium leprae*, and are increasingly growing worldwide (Turenne 2019). While human mycobacterial infections have historically been caused by MTB, currently, NTM is recognized as the main cause of opportunistic mycobacterial infections especially in immunocompromised hosts, and has become a global serious health concern (Turenne 2019; Pennington et al. 2021). NTM can cause various infections, with pulmonary infections being the most frequent, followed by skin and soft tissue infections and disseminated diseases in immunocompromised (HIV-positive in particular) and immunocompetent individuals (Pennington et al. 2021; Tortoli 2009). Therefore, during the last two decades, there has been a special focus on the precise identification of species of NTM by the development of both phenotypic and molecular microbiological diagnostic methods (Varma-Basil et al. 2019).

Today, the use of advanced molecular diagnostic techniques has resulted in the identification of more than 190 mycobacterial species and subspecies (Porvaznik et al. 2017). However, in many low- and middle-income countries such as Iran, where tuberculosis (TB) is endemic, the limited diagnostic potential (both medical and laboratory) remains the main obstacle to determine the real burden of disease and also to detect patients with NTM infections. Another barrier is the wrong treatment of most patients with TB-like infections (Ahmed et al. 2020; Shahraki et al. 2015; Wani et al. 2020). Based on a study from Iran, 15.1% of patients who diagnosed as TB and underwent treatment, were infected by NTM (Nasiri et al. 2018), so due to the differences between the drug therapy of NTM and MTB, the patients faced treatment failure. As the treatment of NTM is species-specific, the rapid and precise identification of NTM at the species level is essential to prevent NTM from being misdiagnosed as multidrug-resistant TB and also to facilitate the early treatment of mycobacteriosis for the most effective outcomes (Pennington et al. 2021; Wani et al. 2020). This would be possible only by employing precise molecular techniques (Chan and Isoman 2013; Tortoli 2014).

Molecular approaches are beneficial tools for the identification and differentiation of Mycobacterial species. DNA probes, PCR–RFLP, real-time PCR, microarray technology, and sequencing, are a number of these methods with high sensitivity and accuracy and a higher speed compared to conventional phenotypic and biochemical methods (Esfahani et al. 2012; Adékambi et al. 2006).The use of gene sequencing technology provides faster and accurate identification of *Mycobacterium spp.* than do phenotypic tests, and many genes have been targeted to detect these bacteria (Cloud et al. 2002). The *16S rRNA, hsp65*, and *rpoB* are the most common genes reported to differentiate mycobacterial species (Turenne et al. 2001; Ringuet et al. 1999; Adekambi et al. 2003). However, an earlier study has documented that the *16S rRNA* is improper for distinguishing closely related species, e.g., pathogenic *M. kansasii* and non-pathogenic *M. gastri*, and unable to make a distinction between MTB and *M. avium* (Vos et al. 2012). The function of the *hsp65* gene in the isolation and differentiation of mycobacterial species is speculated to be superior to the *16S rRNA*; however, the *hsp65* has also not sufficient power to discriminate some closely related species (Kim et al. 2018). The *rpoB* gene, which was first reported in the study of Adekambi et al. (2003), is more extensively used nowadays, compared to other above-mentioned genes. In another investigation conducted by Macheras et al. (2009), a multigenic target approach using five housekeeping gene sequences, including *argH*, *cya*, *glpK*, *gnd*, *murC*, was recommended for those isolates with interspecific composite patterns. Hence, this study was aimed to molecularly identify NTM species isolated from suspected patients by PCR and sequencing of the *rpoB, argH* and *cya* genes and evaluating the efficiency of this three genes markers, in the Khuzestan province of Iran.

## Materials and methods

### Sampling

For this study, 64 clinical isolates of pulmonary origin (sputum and bronchoalveolar lavage [BAL]), were collected from suspected tuberculosis patients who referred to regional TB reference laboratories in Khuzestan province of Iran, from September 2019 to December 2020. The preliminary proposal of the study was approved by the Institutional Ethics and Review Board (Code: IR.AJUMS.REC. 1398. 013) of the Ahvaz Jundishapur University of Medical Sciences, Iran, and the necessary permission for sample collection was granted.

### Culture and biochemical tests

The isolates were cultured on Lowenstein-Jensen (LJ) medium, and incubated at 37 °C. The LJ tubes were examined daily for 30 days to obtain observable colonies. For phenotypic identification of the NTM isolates, standard conventional and biochemical tests, including growth rate, pigment production, acid-fast staining, urease, iron uptake, Tween 80 hydrolysis, heat-stable catalase (pH 7, 68 °C), arylsulfatase, niacin test, and tellurite reduction, according to the standard guidelines provided by the Centers for Disease Control and Prevention (CDC) were performed (Kent 1985). All materials for biochemical tests were purchased from Sigma-Aldrich Corporation (Sigma, St. Louis, MO, USA).

### DNA extraction

For extraction of DNA, the simple boiling method was used as described earlier (Blackwood et al. 2000). In brief, a loopful of bacteria grown on the LJ medium was suspended in one ml of sterile distilled H_2_O containing 5 mm glass beads and vortexed for 2 min for mechanical breakage, and then boiled for 10 min. The quality and quantity of each extracted DNA were checked and measured with the aid of a NanoDrop (ND-1000, Thermo Scientific, DE, USA). The supernatant comprising DNA was kept at −20 °C until use.

### PCR amplification for identification of NTM species by *rpoB, argH* and *cya* genes

For NTM species identification, *rpoB* gene-based PCR was applied. Amplification was performed as a 50 µl reaction mixture, comprising 10 × PCR buffer, 1.5 mM MgCl_2_, 0.2 mM deoxynucleotide triphosphate (dNTPs); 0.2 µM of each primer of mycoF and mycoR primers, as listed in Table 1 (Adekambi et al. 2003), 2.5 U *Taq* polymerase (AMPLIQON, Denmark), and 5 µl of template DNA (10 ng). The PCR program was carried out in a thermocycler (Eppendorf 6333, Hamburg, Germany) as follows: initial denaturation at 95 °C for 5 min, followed by 35 cycles of denaturation at 95 °C for 45 s, annealing at 62 °C for 45 s, and extension at 72 °C for 40 s, with a final extension at 72 °C for 5 min.

**Table 1:** Primer sequences used for PCR amplification and sequence analysis

Gene	Primer	Sequence	Amplified fragment size bp)	Temp (°C)	References
*rpoB*	MYCOF	5^'^-GGCAAGGTCACCCCGAAGGG-3'	750	68	Adekambi et al. 2003
	MYCOR	5^'^-AGCGGCTGCTGGGTGATCATC-3^ʹ^		68	
*argH*	ARGHF	5^ʹ^-GACGAGGGCGACAGCTTC-3'	629	60	Macheras et al. 2009
	ARGHS	5^ʹ^-GTGCGCGAGCAGATGATG-3'		58	
*cya*	ACF	5'-GTGAAGCGGGCCAAGAAG-3'	647	58	Macheras et al. 2009
	ACFR1	5'-AACTGGGAGGCCAGGAGC-3'		60	

About 620-bp and 640-bp fragments of the *argH* and *cya* genes were amplified using sets of primers of *argH* (argininosuccinate lyase), and *cya* (adenylate cyclase), as shown in Table 1 (Macheras et al. 2009). A reaction mixture of 50 µl containing 5 µl of 10 × PCR buffer, 1.5 mM MgCl_2_, 0.2 mM dNTPs, 0.2 µM of each primer, 2.5 U of *Taq* polymerase, and 5 µl of template DNA (10 ng) was prepared. Amplification was done with the following cycling program: initial denaturation at 95 °C for 5 min followed by 35 cycles of denaturation at 95 °C for 40 s, annealing at 59 °C (*argH*)/ 60 °C (*cya*) for 45 s, and extension at 72 °C for 40 s, with a final extension at 72 °C for 5 min.

The PCR products were separated by electrophoresis on a 1.2% agarose gel and stained with the SYBR^®^ Safe DNA Gel Stain (Thermo Fisher Scientific), and by using a gel documentation system (Uvidoc, Jencons Scientific Inc., Cambridge, UK), the DNA bands were visualized (Lee et al. 2012). The size of PCR products were determined using a 100-bp molecular size marker.

### Sequencing of *rpoB, argH* and *cya* genes for detecting NTM species

The amplified PCR products for each isolate, were purified with the DNA Extraction and purification Kit (Bio Basic, Canada), according to the manufacturer’s instructions. An ABI PRISM 7500 Sequence Detection System (Applied Biosystems, Foster City, CA, USA) was utilized to determine the sequences of the products.

The generated sequences of *rpoB*, *argH*, and *cya* for tested isolates were separately analyzed. The GeneBank™ database was employed to analyze and blast the data at the National Center for Biotechnology Information (NCBI) website (http://blast.ncbi.nlm.nih.gov/Blast.cgi). The result of each sequence of NTM isolates was aligned, and a comparison was made with several existing sequences of mycobacterial species in this database. The sequences were aligned using the Clustal W algorithm in the MEGA 11 software. By using the neighbor-joining method, phylogenetic trees were constructed and verified by the maximum likelihood method with 1,000 bootstrap replications. The phylogenetic tree was constructed with MTB and *Nocardia asteroids* as outgroups (Tamura et al. 2021; Saitou and Nei 1987).

## Results

In this study, 64 clinical isolates of NTM, recovered from 50 (78.1%) sputum and 14 (21.9%) BAL were included. These were belonged to 36 (56.25%) female and 28 (43.75%) male suspected tuberculosis patients having fever and cough with a mean age of 54.5 years. Based on the growth rate, the isolates were classified into two groups: rapidly and slowly growing mycobacteria. According to growth characteristics, as well as morphological and biochemical properties, *M. fortuitum*-like isolates (n = 12) were the most frequent isolates, followed by *M. chelonae*-like (n = 11), *M. kansasii* (n = 9), and *M. avium complex* (n = 9) isolates. Among the 64 NTM isolates studied, 51 (79.68%) isolates were identified by phenotypic tests, and the 13 rest were unidentifiable (Table 2). Therefore, complete differentiation of NTM isolates by culture and phenotypic tests was not possible.

**Table 2:** Phenotypic and molecular characteristics of clinical isolates

Isolates no	*rpoB* sequencing	Phenotypic tests	Growth rate	Pigment production	MacConkey agar*	Urease production	Iron uptake	Arylsulfatase (3 days)	68 °C catalase	Nitrate reduction	Niacin production	Tellurite reduction	Tween80 hydrolysis
11	*M. fortuitum*	*M. fortuitum like*	R	N	+	+	+	+	+	+	−	+	+
4	*M. fortuitum*	*M. chelonae*	R	N	+	+	+	+	+	−	−	+	+
1	*M. farcinogenes*	*M. fortuitum like*	R	N	+	+	+	+	+	+	−	+	−
2	*M. simiae*	*M. scrofulaceum*	S	Y/Sc	−	−	−	+	+	−	−	+	−
2	*M. thermoresistibile*	*M. gordonae*	S	Y/Sc	+	+	+	−	−	+	−	+	+
6	*M. abscessus*	*M. chelonae*	R	N	+	+	+	−	+	−	−	+	−
5	*M. simiae*	*M. simiae*	S	Y/P	−	−	−	−	+	−	+	+	−
9	*M. kansasii*	*M. kansasii like*	S	Y/P	−	+	−	−	+	+	−	+	+
3	*M. yangoense or M. chimerae*	*M. avium complex*	S	N	+	−	−	−	−	−	−	+	−
5	*M. yangoense or M. chimerae*	*M. avium complex*	S	N	+	−	−	−	+	−	−	+	−
2	*M. elephantis*	*Mycobacterium sp.*	S	Y/P	−	+	−	+	+	+	−	+	+
3	*M. kansasii*	*Mycobacterium sp.*	S	Y/Sc	−	+	−	−	+	+	−	−	+
4	*M. abscessus*	*Mycobacterium sp.*	R	N	+	+	+	−	−	−	−	+	−
4	*M. abscessus*	*Mycobacterium sp.*	R	N	+	+	+	+	−	−	−	+	−
1	*M. chelonae*	*M. chelonae*	R	N	+	+	+	+	+	−	−	+	+
1	*M. intracellulare*	*M. avium complex*	S	N	+	−	−	−	+	−	−	+	−
1	*M. gordonae*	*M. gordonae*	S	Y/Sc	+	+	+	−	−	+	−	+	+

*R* Rapid, *S* Slow, *N* Non-pigmentation, *Y* Yellow, *Sc* Scotochromogen

^*^Growth on MacConkey agar

The results of *rpoB* sequence analysis revealed that 56 clinical isolates were identified in 10 species of NTM, including *M*. *fortuitum* (15 isolates), *M. abscessus* (14 isolates), *M. kansassi* (12 isolates), *M. simiae* (7 isolates), *M. thermoresistible* (2 isolates), *M. elephantis* (2 isolates), *M. farcinogenes*, *M. gordonae*, *M. intracellular* and *M. chelonae*, one isolate each (Tables 2), and the rest 8 isolates showed ambiguous results and were not identified accurately by the *rpoB* gene. To evaluate the efficacy of *argH* and *cya* genes for the detection of various species of NTM, we selected at least one isolate of each species, that was previously identified by the *rpoB* sequencing. Among the 8 obscure isolates not identified by *rpoB,* 5 isolates showed 100% similarity to *M. yongoense*, and the remaining 3 isolates were also identified as *M. chimera* using *argH* and *cya* paritial sequence analysis.

The clinical isolates of NTM11, NTM46, NTM7, and NTM73 representing the *M. abscessus* strains were studied with *argH* and *cya* genes. All four *M. abscessus* isolates were confirmed as *M. abscessus subsp. abscessus* by amplifying and sequencing the *argH* gene. However, in the analysis of *cya* gene, NTM11 and NTM46 isolates belonged to *M. abscessus subspecies abscessus*, with 100% similarity to the reference isolate *M. abscessus* strains YMC11_01, and NTM73 and NTM7 isolates belonged to *M. abscessus subspecies massiliense*, with 99.93% similarity to *M. abscessus subsp massiliense* strain YMC1119. The rest of the isolates showed similar results to those achieved from the *rpoB* gene sequencing (Table 3).

**Table 3:** Results of *cya* and *argH* sequencing for identification of NTM species and the similarity percentage of each isolate with the reference strain

Tested isolates	*Cya* sequencing	Similarity (%)	*argH* sequencing	Similarity (%)
NTM361,363,411,419,359 (*M. yongonense or M. chimaera)*	*M. yongonense*	100	*M. yongonense*	100
NTM 18,30,90 (*M. yongonense or M. chimaera)*	*M. chimaera*	100	*M. chimaera*	100
NTM 329 *(M. intracellulare)*	*M. intracellulare*	99.43	*M. intracellulare*	99.46
NTM108,112 (*M. kansasii)*	*M.kansasii*	100	*M.kansasii*	100
NTM304 (*M.gordonae)*	*M.gordonae*	99.93	*M.gordonae*	96.94
NTM409 *(M.chelonae*)	*M.chelonae*	100	*M.chelonae*	100
NTM46,11 (*M.abscessus)*	*M.abscessus subsp. abscessus*	100	*M.abscessus subsp. abscessus*	100
NTM7,73 (*M.abscessus*)	*M.abscessus subsp.massiliense*	99.93	*M.abscessus subsp. abscessus*	98.38
NTM74,34 (*M.Simiae*)	*M.simiae*	100	*M.simiae*	98.56
NTM35 *(M. elephantis*)	*M.elephantis*	100	*M.elephantis*	98
NTM87,12,360 (*M.fortuitum)*	*M.fortuitum*	100	*M.fortuitum*	99.28
NTM410 (*M.farcinogenes*)	*M.farcinogenes*	100	*M.farcinogenes*	99.28
NTM420,32 (*M.thermoresistible)*	*M.thermoresistible*	100	*M.thermoresistible*	100

**Fig. 1:**
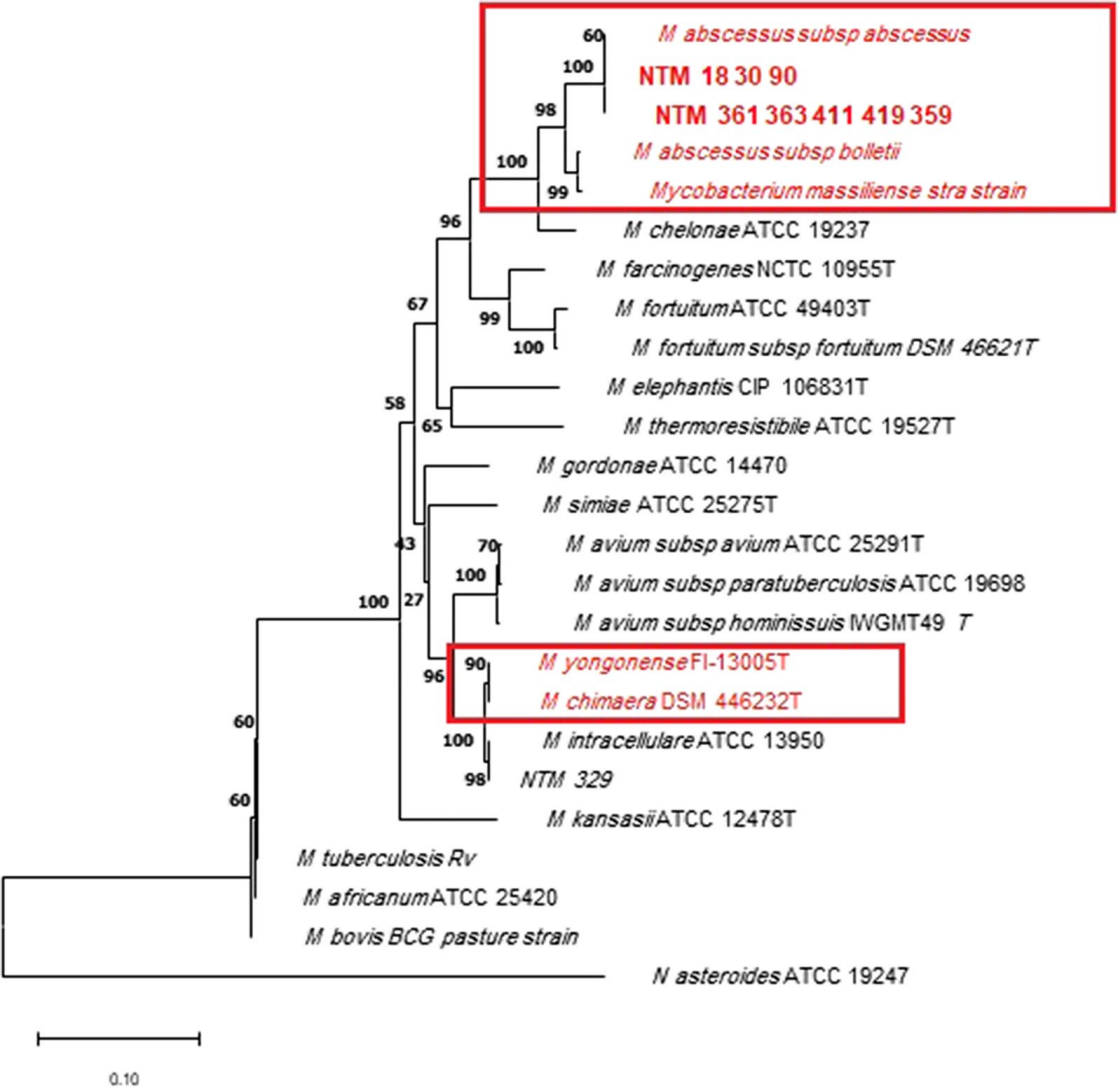
Phylogenetic relationship (evolutionary tree) of clinical isolates of nontuberculous Mycobacteria based on *rpoB* gene sequencing (the maximum likelihood tree generated using the Tamura 3-parameter model in MEGA11 software). Boxes are represented of isolates that could not be identified by *rpoB* sequencing

**Fig. 2:**
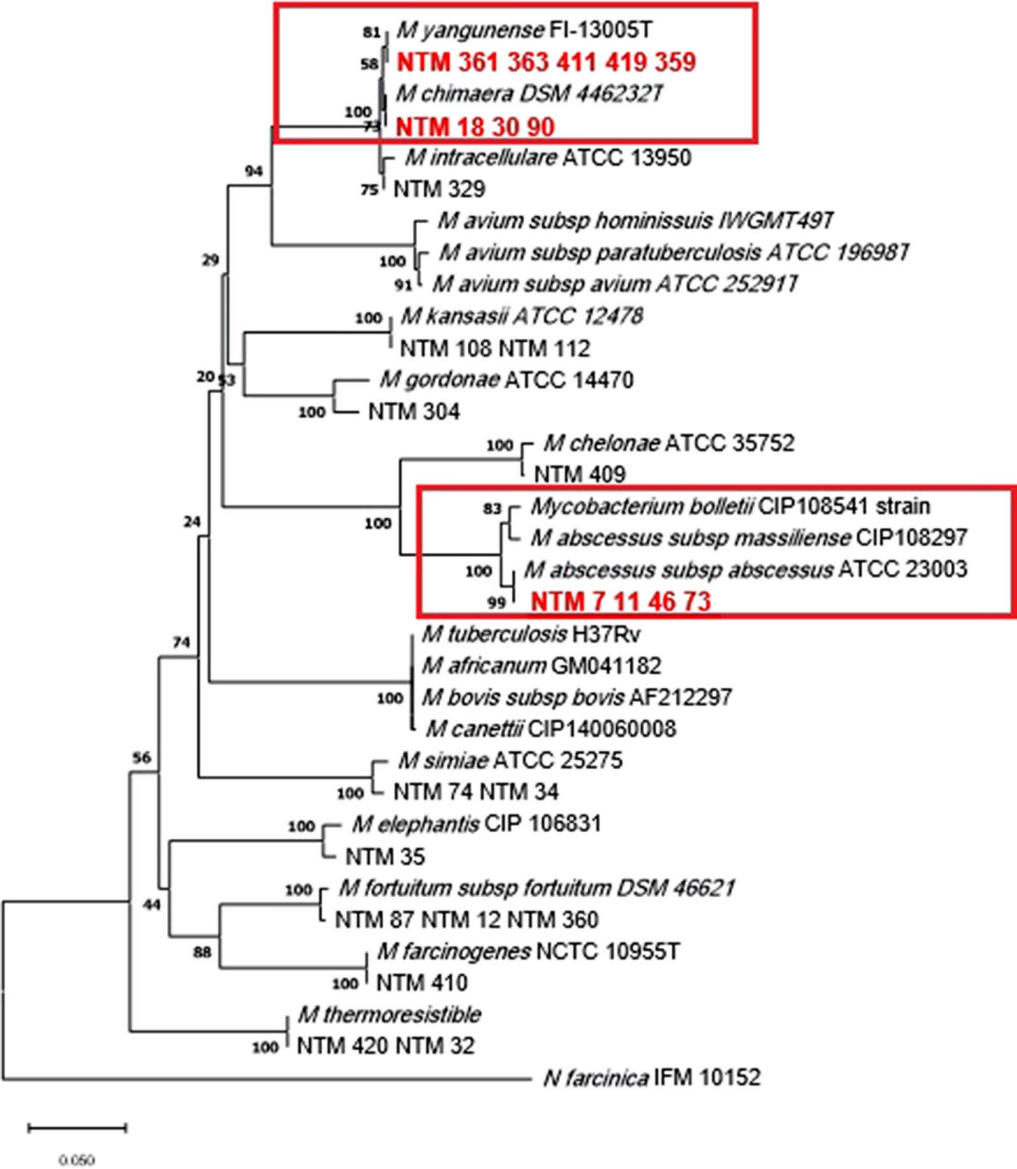
Phylogenetic relationship (evolutionary tree) of clinical isolates of nontuberculous Mycobacteria based on *argH* gene sequencing (the maximum likelihood tree generated using the Tamura 3-parameter model in MEGA11 software). Boxes are represented of isolates that could be identified by *argH* sequencing

**Fig. 3:**
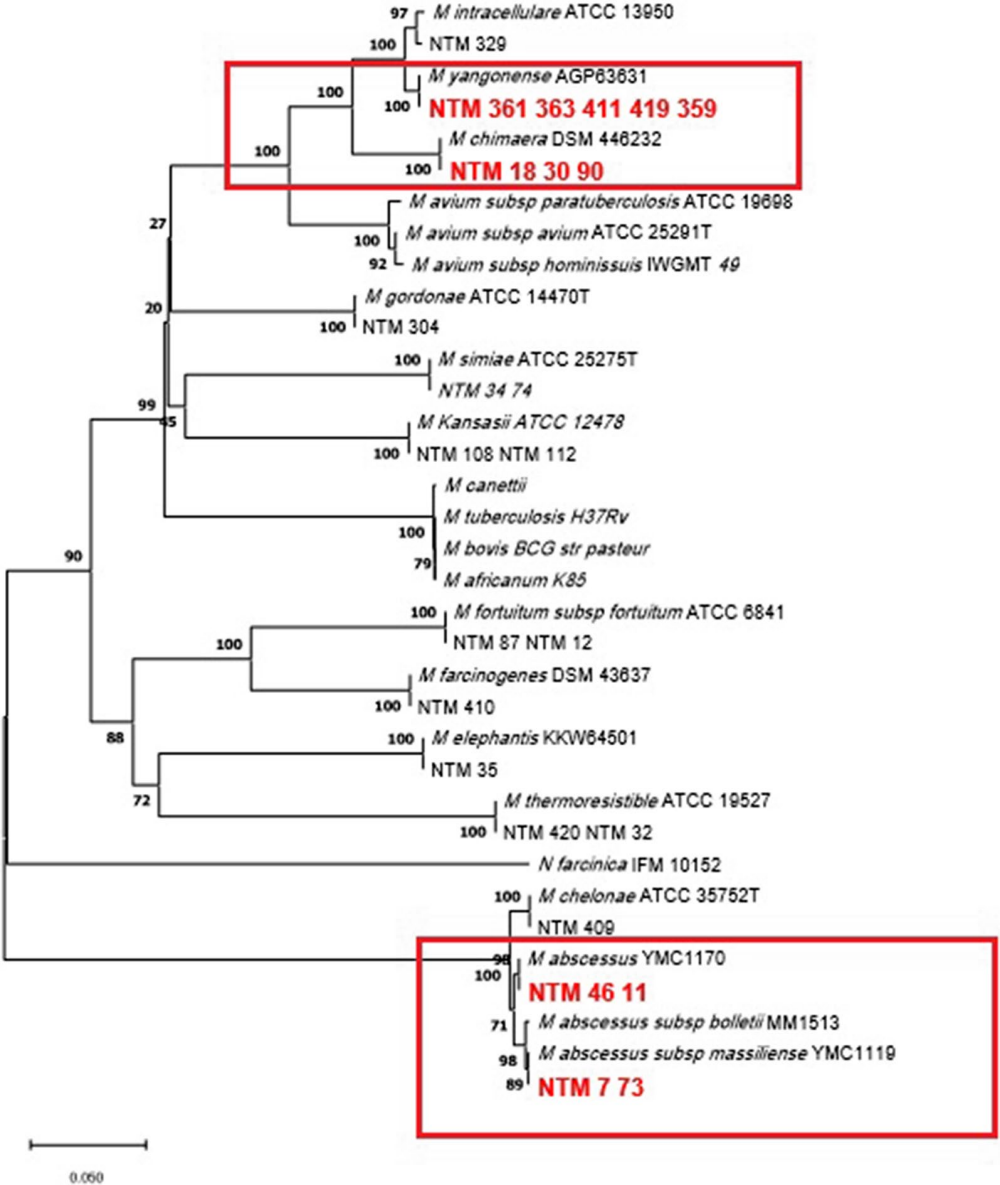
Phylogenetic relationship (evolutionary tree) of clinical isolates of nontuberculous Mycobactera based on *cya* gene sequencing (the maximum likelihood tree generated using the Tamura 3-parameter model in MEGA11 software). Boxes are represented of isolates that could be identified by *cya* sequencing

Dendrogram showing the phylogenetic relationship of NTM isolates based on *rpoB*, *argH* and, *cya* genes sequencing are presented in Figs. 1, 2, 3.

## Discussion

Today, due to the increase in the number of immunocompromised patients worldwide (such as AIDS and cancer cases, transplant recipients, and users of immunosuppressive drugs), we are facing a rise in NTM infections (Pennington et al. 2021). As mentioned earlier, in countries such as Iran, where TB is endemic, the NTM infections are misdiagnosed as TB and reported as MTB (Nasiri et al. 2018) Since most NTMs are inherently resistant or semi-sensitive to standard anti-TB drugs, thus, the accurate detection of NTM species in related infections is of importance for choosing an appropriate treatment plan. As phenotypic (conventional) methods of identifying Mycobacteria are time-consuming, and fail to detect some species accurately, applying complementary methods such as molecular techniques are necessary (Ahmed et al. 2020; Shahraki et al. 2015; Wani et al. 2020; Nasiri et al. 2018; Chan and Isoman 2013; Tortoli 2014).

The present study revealed that conventional methods are insufficient to detect different NTM species, and only 36 isolates (56.25%) were correctly identified by these methods, compared with the results of molecular techniques. In our study, the *rpoB* gene sequencing could accurately identify 56 isolates, and the remaining isolates were precisely distinguished by sequencing of the *argH* and *cya* genes. Considering these data, we come to the conclusion that the sequence-based methods are capable of identifying various Mycobacterium species more precisely and rapidly than phenotypic methods, as represented in several studies (Kim and Shin 2018; Khosravi et al. 2016; Shahraki et al. 2017). Moreover, both *argH* and *cya* genes comprise high discriminatory power to identify and distinguish closely related species.

Our study was coordinated with Macheras et al. (2009) study, regarding the capability of *argH* and *cya* genes. In their study, the isolates that could not be identified by the *rpoB* and *sodA* gene sequencing method, were readily separated and detected by sequencing of housekeeping genes *argH* and *cya*. However they showed discordance between *rpoB* sequencing and other genes for discrimination of *M. abscessus* group with interspecific composite patterns. Although, Kim et al. (2019), also suggested the high efficiency of these two genes in distinguishing varying subspecies of *M. abscessus*.

The main discrepancy between our study and the two other mentioned studies is that their study was conducted only on fast-growing isolates of *M. abscessus*, but we analyzed the efficiency of *argH* and *cya* genes to detect not only the *M. abscesses* but also the fast- and slow-growing species of Mycobacterium. We showed that sequencing of these two genes contributed to the identification and differentiation of all the isolates in this study, and slow-growing bacteria such as *M. avium* complex. In another study conducted by Macheras et al. (2014), it was explored that the *rpoB* gene was unable to discriminate the three subspecies of *M. abcsessus*, i.e. *abscessus*, *bolleti*, and *massiliense*, but sequencing the housekeeping genes of *argH*, *cya*, *glpK*, *gnd*, *murC*, *pta*, *and purH* could identify them. The results of our study also confirmed this finding and reflected that the use of *cya* sequencing is highly efficient in differentiating these subspecies.

According to a study by Khosravi et al. (2018), *M. fortuitum* isolated from different provinces of Iran was the most prevalent NTM in the country; however, its infection was misdiagnosed as TB in many patients. Therefore, the exact detection of this fast-growing species is crucial. In the current study, the *cya* gene had the best efficiency in identifying this species because of its high similarity (100%) with the reference strain of *M. fortuitum subsp fortuitum* ATCC 6841, which could distinguish this species. A recent case report by Rodriguez et al. (2021), uncovered the possibility of simultaneous infection with Mycobacteria and COVID-19. In this survey, a patient with both multiple myeloma and COVID-19 was also infected with *M. abscessus*. This case report highlights the importance of examining COVID-19 co-infections to provide targeted treatment, since treatment plans for COVID-19 and NTM infections are quite different, while the symptoms are the same. Likewise, when patients appear to be primarily infected with COVID-19, it is important to evaluate other infections because an appropriate antibiotic treatment can change the outcome of treatment. This information adds to the value of *argH* and *cya* genes, especially inaccurate identification of *M. abscessus* subspecies. It is also important to note that the outbreak of the COVID-19 virus has necessitated fundamental adjustments to the healthcare system (Zhu et al. 2020). Therefore, research on bacterial infections with COVID-19 is ongoing, though there is scarce data. The results of study of Rawson et al. (2020), revealed that 18% of COVID-19 patients experienced bacterial/fungal infections while hospitalized. Taken together, NTM infections in patients with COVID-19 are suggested to be investigated in future research projects.

Our work, similar to some other studies, had some limitations. First, the number of NTM isolates was low, and to justify the role of *cya* and *argH* genes sequencing, we need to extend the duration of our future studies to have more isolates. Second, owing to the general weakness of the country’s health system in recording and tracking the exact demographic data of patients, most of the clinical information of patients are incomplete, and this project was no exception, and the third limitation was the financial inability to examine other housekeeping genes in identifying different species of Mycobacterium. Further studies on sequencing additional housekeeping genes for detecting varied Mycobacterium species help to recognize the potential genes target for the identification of NTM infections.

In conclusion, by summarizing the results of this study and previous studies, we can express that *rpoB* sequencing is still a supreme diagnostic method. Furthermore, due to the high ability of *argH* and *cya* genes to confirm and differentiate similar species, these two genes can be employed for accurate identification and effective treatment of clinical infections, even in future epidemiological studies. Further clinical trials are required to support our findings.

## Data Availability

All data generated or analyzed during this study are included in the present published article and its supplementary information file.

## Abbreviations

NTM: Nontuberculous mycobacteria
MTB: *Mycobacterium tuberculosis*
HIV: Human immunodeficiency virus
BAL: Bronchoalveolar lavage
PCR–RFLP: Polymerase chain reaction-restriction fragment length polymorphism
LJ: Lowenstein-Jensen
CDC: Centers for disease control and prevention
dNTP: Deoxynucleotide triphosphate
NCBI: National Center for Biotechnology Information
